# Supplementary material for: Is It Useful to Repeat Blood Cultures in Endocarditis Patients? A Critical Appraisal
Source: Diagnostics (Basel). 2024 Jul 22;14(14):1578. doi: 10.3390/diagnostics14141578 (PMC11276044; doi:10.3390/diagnostics14141578)
Supplement: Supplementary file 1 [file diagnostics-14-01578-s001.zip › diagnostics-3048324-supplementary.pdf]

Supplemental file, Pubmed search query.

Query 1 on the incidence of and association of bacteremia with outcome in endocarditis patients

(Humans OR Female OR Male) AND (Endocarditis, Bacterial / blood OR Endocarditis, Bacterial / microbiology ) AND (Culture Techniques / statistics & numerical data* OR Bacteriological Techniques OR Drug Monitoring / methods* OR Blood Culture OR Bacteremia / blood* OR Bacteremia / microbiology OR Endocarditis, Bacterial / microbiology) AND (Case-Control Studies OR Cohort Studies OR Retrospective Studies OR Prospective Studies OR Multivariate analysis) AND (Bacteremia / mortality OR Endocarditis, Bacterial / mortality OR Survival Analysis OR Treatment Outcome OR Prognosis)

This search provided 478 results, with 45 publications reviewed and 10 studies selected. There were 12 studies added from reviewing cited references from these publications. The final review also included 3 endocarditis guidelines and used 31 publications for the first query.

Query 2 on the influence of antibiotic therapy duration on valve culture positivity at the time of surgery.

(Humans OR Female OR Male) AND (Endocarditis, Bacterial / blood OR Endocarditis, Bacterial / microbiology ) AND ((Anti-Bacterial Agents / therapeutic use OR Anti-Bacterial Agents / administration & dosage* OR Endocarditis, Bacterial / drug therapy) OR (Cardiac Surgical Procedures / methods OR Cardiac Surgical Procedures / standards*)) AND (Case-Control Studies OR Cohort Studies OR Retrospective Studies OR Prospective Studies) AND (Heart Valves / microbiology* OR Treatment Outcome)

This search provided 156 results, with 5 publications reviewed and 2 studies selected. There were 7 studies added from reviewing cited references from these publications. The final review used 8 publications for the second query.
